# Mechanical allodynia

**DOI:** 10.1007/s00424-014-1532-0

**Published:** 2014-05-22

**Authors:** Stéphane Lolignier, Niels Eijkelkamp, John N. Wood

**Affiliations:** Molecular Nociception Group, Wolfson Institute for Biomedical Research, University College London, London, WC1E 6BT UK

**Keywords:** Mechanical allodynia, Mechanotransduction, Piezo2, cAMP, Neuropathic pain

## Abstract

Mechanical allodynia (other pain) is a painful sensation caused by innocuous stimuli like light touch. Unlike inflammatory hyperalgesia that has a protective role, allodynia has no obvious biological utility. Allodynia is associated with nerve damage in conditions such as diabetes, and is likely to become an increasing clinical problem. Unfortunately, the mechanistic basis of this enhanced sensitivity is incompletely understood. In this review, we describe evidence for the involvement of candidate mechanosensitive channels such as Piezo2 and their role in allodynia, as well as the peripheral and central nervous system mechanisms that have also been implicated in this form of pain. Specific treatments that block allodynia could be very useful if the cell and molecular basis of the condition could be determined. There are many potential mechanisms underlying this condition ranging from alterations in mechanotransduction and sensory neuron excitability to the actions of inflammatory mediators and wiring changes in the CNS. As with other pain conditions, it is likely that the range of redundant mechanisms that cause allodynia will make therapeutic intervention problematic.

## Summary

Mechanical allodynia (‘other pain’) is a painful sensation caused by innocuous stimuli like light touch. Unlike inflammatory hyperalgesia that has a protective role, allodynia has no obvious biological utility. Allodynia is associated with nerve damage in conditions such as diabetes and is likely to become an increasing clinical problem. Unfortunately, the mechanistic basis of this enhanced sensitivity is incompletely understood. In this review, we describe evidence for the involvement of candidate mechanosensitive channels such as Piezo2 and their role in allodynia, as well as the peripheral and central nervous system mechanisms that have also been implicated in this form of pain. Specific treatments that reverse mechanical allodynia could be very useful if the cell and molecular basis of the condition could be determined. There are many potential mechanisms underlying this condition ranging from alterations in mechanotransduction and sensory neurons excitability to the actions of inflammatory mediators and wiring changes in the CNS. As with other pain conditions, it is likely that the range of redundant mechanisms that cause allodynia will make therapeutic intervention problematic.

## Introduction

**Fig. 1:**
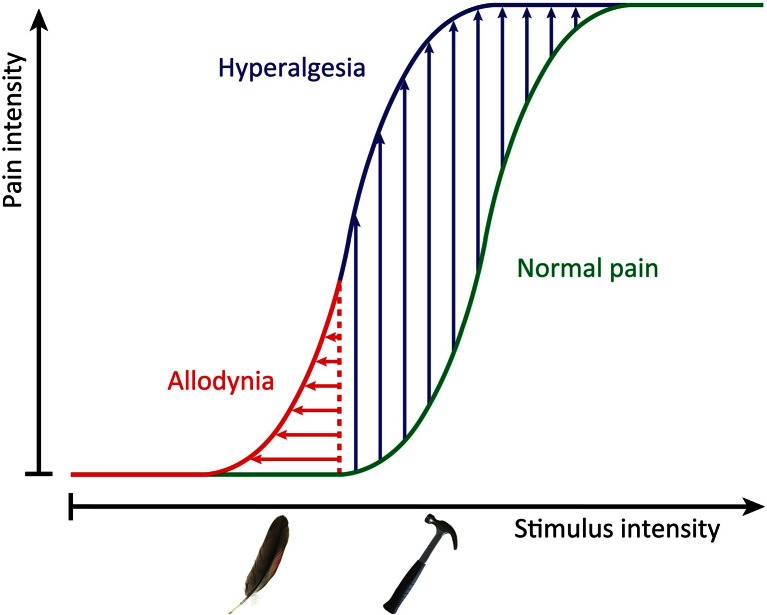
Sensitization to pain. This graphical representation of the shift in pain thresholds during a pain state shows both enhanced response to noxious, normally painful, stimuli (hyperalgesia) and pain triggered by non-noxious stimuli (allodynia) like the gentle brush of the skin. These two painful states do not always coexist, and it is increasingly apparent that they are driven by distinct mechanisms in different sets of sensory neurons

Pain is a vast and increasing problem, affecting around a fifth of the population. This problem is exacerbated by ageing populations with conditions such as diabetes and osteoarthritis, who suffer an even higher incidence of ongoing pain. Strikingly, the drug industry has made little progress in developing new classes of analgesics, and many companies have given up the struggle, making the development of useful treatments even less likely. Mechanical allodynia is a condition where pain caused by innocuous stimuli like the touch of clothing may be debilitating, but the condition is little addressed in terms of drug development because other pain syndromes are more common. The phenotypic distinction between allodynia and hyperalgesia is shown schematically in Fig. 1.

There have been some recent advances in understanding the cell and molecular basis of mechanical hyperalgesia. For example, a critical role for NGF in damaged tissues has been linked to mechanically evoked pain in osteoarthritis, and this pain can be reversed in man by the application of neutralising anti-NGF monoclonal antibodies [21]. At the same time, the expression of mechanosensitive channels on the surface of cultured sensory neurons has been shown to be upregulated by NGF through a transcriptional mechanism, linking sensory neuron mechanotransducing channels functionally with enhanced mechanical pain [5]. This observation has led to attempts to characterise intrinsically mechanosensitive ion channels on sensory neurons and examine their role in mechanotransduction, hyperalgesia and allodynia.

## Peripheral mechanisms linked to mechanical allodynia

Mechanotransducing channels expressed by sensory neurons in culture have been characterised electrophysiologically using a technique developed by Jon Levine [26]. Mechanosensitive sensory neurons can be divided into subpopulations according to the mechanically activated current they generate in response to membrane distension. Rapidly adapting neurons involved in touch and proprioception are known to display a low threshold current with fast inactivation kinetics, consistent with their tonic response to mechanical stimuli. Mechano-nociceptors in turn show a mixed repertoire of rapidly, intermediately and slowly adapting currents with higher activation thresholds, accordingly to the high intensity of the stimuli to be detected [6]. It is still unknown whether the rapidly adapting currents found in nociceptors involve the same channel(s) as the rapidly adapting current present in low threshold mechanoreceptors. Evidence of a role for the rapidly adapting channels Piezo2 and TRPC3/C6 in non-noxious mechanotransduction by touch sensitive cells, as well as in light touch sensitivity in vivo, has been obtained [10, 34].

When considering mechano-nociception, the slowly adapting current found in sensory neurons is of particular interest. Its slow inactivation kinetics is coherent with the phasic (slowly adapting) afferent response coded by mechano-nociceptors. More direct evidence of its contribution to mechano-nociception has also been established. The conotoxin NMB-1, shown to be a specific blocker of the slowly adapting current in sensory neurons of the dorsal root ganglia, increases mechanical pain thresholds without affecting light touch sensitivity in vivo [8]. Exposure to botulinum toxin has also been shown to chronically, but reversibly, impair mechano-nociception in humans. In parallel, the number of sensory neurons expressing a slowly adapting, mechanically activated, current is decreased in mice in vitro when these neurons are cultured and exposed to botulinum toxin [30]. The molecular identity of the channel or protein complex responsible for the slowly adapting current remains unknown. The TRPA1 channel has been found necessary for the existence of such a current in a subset of peptidergic sensory neurons [40] and TRPA1 blockade reduces the firing of sensory fibres in response to noxious mechanical stimuli [17]. In vivo, Trpa1^−/−m^ice were also shown to have a higher mechanical pain threshold when analysed in the von Frey test [20, 32]. These are convincing pieces of evidence for a relatively direct role of TRPA1 in the transduction of noxious mechanical stimuli. However, TRPA1 fails to generate mechano-gated currents when expressed in a heterologous system, showing that it is not the mechanotransducer itself or that one or more accessory proteins are required for TRPA1 to be opened by membrane stretch [35, 40]. As TRPA1 is also expressed in keratinocytes that are in close contact with nerve terminals in the skin, an indirect contribution of TRPA1 to noxious mechanotransduction cannot be excluded.

Furthermore, stretch-activated channels have to be considered as part of the wider transduction machinery involved in specific tuning of cell excitability. For example, the Kv1.1 potassium channel, whose voltage-gated activation has recently been shown to be facilitated by membrane stretch [13], has current kinetics that allows it to counter the depolarization induced by the activation of the slowly-activating, but not the rapidly-activating, mechano-gated channels present in nociceptors. As a consequence, Kv1.1 blocking increases mechanically induced depolarization in nociceptors, as well as their firing threshold in response to mechanical stimuli, and Kv1.1 knockout in mice are found to be hypersensitive to mechanical stimuli in vivo. Other potassium channels like TREK-1 and TRAAK, from the K2P family, have been shown to be important for mechanical pain [2, 29]. However, they are not specific for this modality and are rather involved in setting the overall level of nociceptor excitability, whatever their function may be.

Targeting the channel responsible for the slowly adapting current, characteristic of noxious mechanotransduction, could perhaps contribute to reducing pain in people suffering from mechanical pain hypersensitivity, but rather than inhibiting noxious mechanosensation, which could eventually lead to new injuries or to the worsening of the original problem, for example by aggravating a joint condition, we should aim for the re-establishment of normal pain sensitivity. This is very well illustrated by the effect of the anti-NGF treatment Tanezumab in osteoarthritic patients [21]. NGF is known to promote the over-expression of mechanosensitive channels underlying rapidly as well as slowly adapting currents, and PKC activation by inflammatory factors such as bradykinin addresses the newly produced channels into the membrane [5]. Neutralising NGF with Tanezumab has proven successful in reducing joint pain, however, the induced mechanical analgesia has also been linked in a small number of cases with rebound inflammation and joint failure in some of the people treated with anti-NGF monoclonal antibody [44].

Details about the mechanisms leading to mechanical allodynia, including what are the final effectors, and which neuronal subtypes are involved, provide a complex picture. The depletion of Nav1.8-positive sensory neurons in mice by crossing animals carrying a floxed-stop upstream of the diphtheria toxin A gene with Nav1.8-Cre mice reverses Freund’s complete adjuvant-induced mechanical hyperalgesia [1]. However, genetic ablation of Nav1.8-positive neurons was ineffective in reducing neuropathic mechanical allodynia induced by spinal nerve transection. This shows that different mechanisms, involving different populations of sensory neurons, can cause mechanical hypersensitivity. We made a similar observation on allodynia associated with neuropathic pain using two models of traumatic neuropathic pain, induced by spinal nerve transection and chronic constriction of the sciatic nerve. In the first case, Nav1.7 conditional knock-out in the whole dorsal root ganglion was sufficient to alleviate mechanical allodynia, but the Nav1.7 knock-out had to be extended to sympathetic neurons to abolish allodynia in a sciatic chronic constriction injury [27]. The interplay between the sympathetic nervous system and sensory neurons seems to be an important factor in many types of neuropathic pain. However, there are examples of mechanical allodynia (e.g. bone cancer pain) that occur in the absence of Nav1.8-positive nociceptors, or sodium channel Nav1.7 (considered essential for human pain) whose mechanisms are completely obscure [27].

**Fig. 2:**
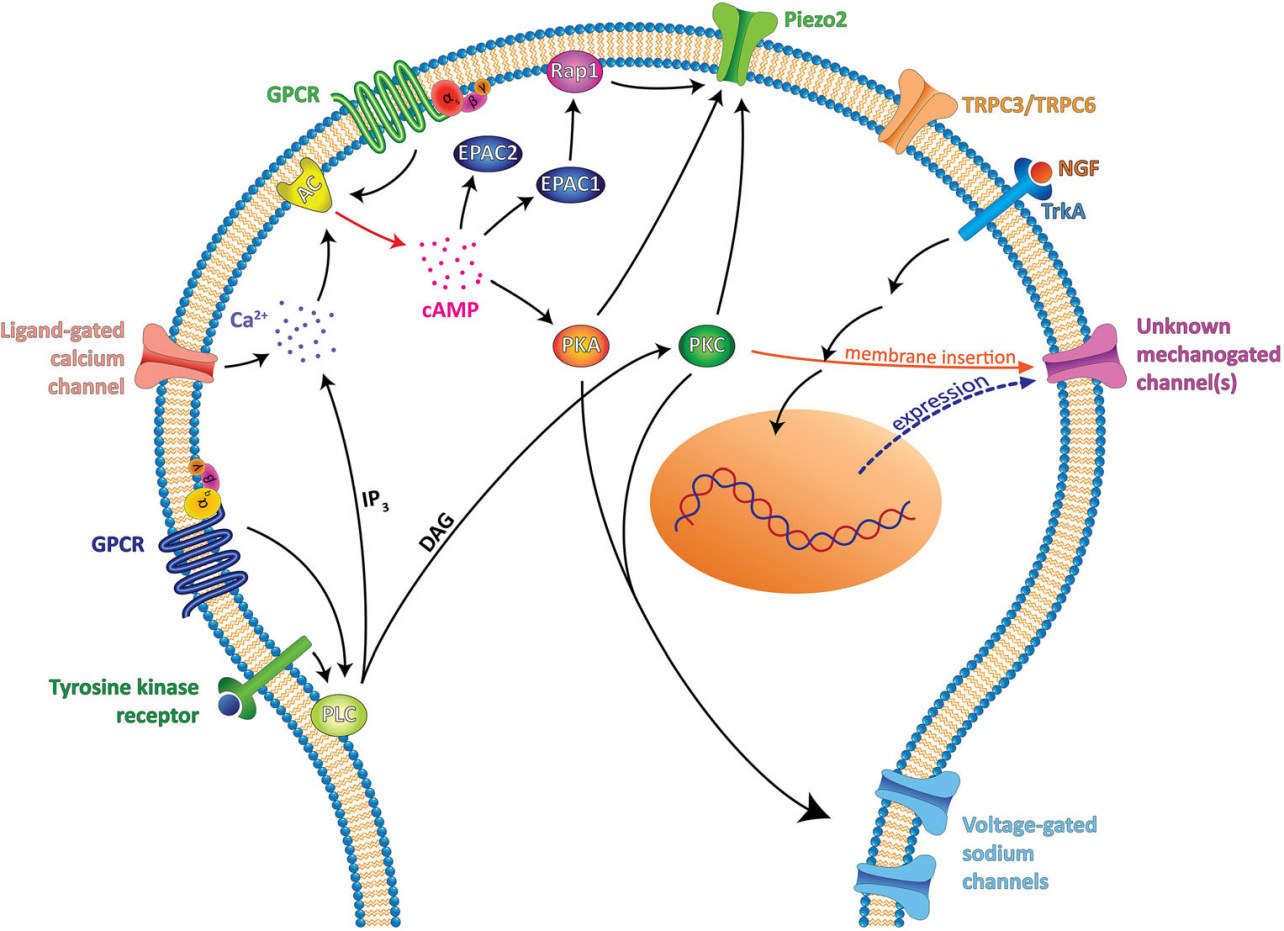
Signalling pathways for the sensitization of mechanosensitive sensory neurons. Different but interconnected pathways have been shown to contribute to the sensitization of mechanosensitive neurons, leading to allodynia. PKA and PKC are known to be important for neuronal excitability, notably through voltage-gated sodium channel modulation [12, 39], but more recently, they were also found to have an effect on mechanotransduction directly. Indeed, the low threshold mechano-gated channel Piezo2 has been shown to be positively modulated by PKC, PKA [9] and by the cAMP sensor EPAC1 [10]. EPAC1, but not EPAC2, enhances Piezo2 current by activating the G protein Rap1 when activated by cAMP. cAMP increases can be induced directly by the activation of GPCR coupled to Gs proteins (PGE_2_ receptor EP_2_, histamine H2 receptor, CALCRL receptor for CGRP, serotonin receptor 5-HT_4_…) or indirectly via a calcium increase, induced either by an ion channel (TRPs, ASICs, P2Xs…) or by IP_3_-mediated calcium increase following either activation of a PLC coupled tyrosine kinase receptor (e.g. neurotrophin receptors) or activation of a GPCR coupled to Gq proteins (PGE_2_ receptor EP_1_, histamine receptor H1, serotonin receptor 5-HT_2_…). PLC activation will also result in DAG-mediated activation of PKC, which has also been shown to positively regulate Piezo2 channels [9]. However, PKA and PKC activation were found to have no effect on the human Piezo2 channel [10]. Therefore, further work is needed to clarify Piezo2 modulation by these signalling pathways. TRPC3 and TRPC6 channels were also found to be involved together in the generation of a low threshold mechanically activated current and to be essential for normal touch perception [34]. It is not known whether these channels are subject to the same positive regulation as Piezo2. Mechano-gated channels producing rapidly, intermediately and slowly adapting currents, yet to be identified, are also positively regulated by NGF and PKC [5]. In sensory neuron cultures, TrkA activation by NGF leads to transcription of new channels, and activated PKC promotes the insertion of the channels into the membrane to increase peak currents [2]

Much effort has been put into elucidating the specific signalling cascades involved in the sensitization of sensory neurons by immune mediators, consequently leading to the development of hyperalgesia. Increasing evidence suggests that sensitization of both transduction and neurotransmission in nociceptors is a major cause of inflammatory hyperalgesia [16, 45]. For example, inflammatory mediators have profound effects on the heat transducer TRPV1 and voltage-gated sodium channels present in sensory neurons. However, evidence for the regulation of mechanical transduction in allodynic states is just starting to emerge. The known pathways involved are summarised in Fig. 2. The first intracellular messenger associated with sensory neuron sensitization was cAMP. Intradermal injections of cAMP analogues induce profound mechanical hyperalgesia (reduced response thresholds to noxious mechanical stimuli measured with the Randall-Selitto test) but also increased responses to innocuous mechanical stimuli (as measured with von Frey hairs), that is characteristic of allodynia [36]. cAMP signalling has been primarily implicated in the development of pain hypersensitivity in an inflammatory state as many inflammatory mediators signal through G protein-coupled receptors that are coupled with G proteins that activate (Gs) adenylate cyclase, whilst other inflammatory mediators may cause an increase in intracellular calcium that activates calcium-sensitive adenylate cyclases. Pharmacological and genetic evidences also suggest a role for cAMP in the development of mechanical allodynia in rodent models of neuropathic pain [18, 22, 23]. For example, the development of mechanical allodynia is severely impaired in models of neuropathic pain in mice deficient for adenylate cyclase 5 [18]. Several groups have used pharmacological and genetic tools to identify whether the downstream cAMP sensor PKA plays a role in mechanical allodynia in neuropathic pain conditions. However, these studies did not show any evidence for PKA involvement in such a condition [24, 46]. Yet strong evidence exists for a role of PKA in the development of inflammatory mechanical hyperalgesia and enhanced neuronal excitability has been linked to PKA mediated phosphorylation of sodium channels [16].

Although increases in intracellular cAMP have been considered to be equivalent to the activation of the cAMP sensor protein kinase A (PKA), in 2001, the Bos group identified a separate pathway activated by cAMP, the family of exchange factors activated by cAMP (Epac). Activation of Epac leads to subsequent activation of a small G protein: Rap1, that is upstream of various effector proteins, including adaptor proteins that affect the cytoskeleton, regulators of G proteins of the Rho family, phospholipases and protein kinases [14]. In non-peptidergic isolectin B4 positive sensory neurons, Epac signalling mediates cAMP-to-PKCε signalling, leading to inflammatory hyperalgesia [15]. We recently showed that injection of a specific Epac activator also induces hypersensitivity to innocuous mechanical stimuli that is independent of Nav1.8 nociceptors [10], normally associated with the development of inflammatory hyperalgesia [1]. In contrast, activation of the PKA signalling cascade by intradermal injections of a specific PKA agonist also results in reduced mechanical thresholds, yet this mechanical hypersensitivity was completely dependent on Nav1.8 neurons. In Epac1 knockout mice, nerve damage induced mechanical allodynia is impaired, indicating an important role for this cAMP sensor in the development of mechanical allodynia. Interestingly, in chronic inflammatory pain, knockdown of Epac1 during already developed persistent mechanical hypersensitivity also reduces the symptom, whilst acute transient inflammatory hyperalgesia is unaffected in Epac1 knockout mice [42].

In vitro activation of the Epac signalling cascade sensitises large diameter neurons that express rapidly adapting mechanically activated currents [10] and that have been linked to touch sensitivity [43]. The Piezo protein family that includes Piezo1 and Piezo2 has been shown to mediate some of the rapidly adapting currents in sensory neurons [5]. In a heterologous expression system, activation of the cAMP sensor Epac1 enhances Piezo2 currents, whilst specific activation of PKA using 6-Bnz-cAMP, activation of PKC using PMA or activation of Epac2 by 8-pCPT does not affect Piezo2 sensitivity [10]. Thus, cAMP-to-Epac1 signalling would specifically sensitises Piezo2 current. Inflammatory mediators are also capable of sensitising Piezo2 sensitivity. The algogenic peptide bradykinin produced during inflammation enhances Piezo2 current in a heterologous expression system [9]. This effect is blocked by the concomitant use of PKA and PKC inhibitors, and in parallel, the use of either a cAMP analogue or a PKC activator were shown to enhance Piezo2 current. In vivo, we found that sensitization of Piezo2 by Epac1 plays a role in the development of mechanical allodynia. Partial antisense knockdown of Piezo2 in the dorsal root ganglia of mice attenuates mechanical allodynia in two different model of nerve injury. In addition, mechanical allodynia induced by intraplantar injection of a specific Epac agonist is attenuated by Piezo2 knockdown [10]. These findings highlight that the tuning of Piezo2 current by the cAMP-Epac1 signalling cascade plays an important role in the dysregulation of mechanotransduction leading to mechanical allodynia.

An important question that arises is whether in chronic neuropathic pain conditions, continuous driving forces such as elevated levels of cAMP sustain the sensitization of mechanotransduction leading to allodynia. Recently, a specific Epac antagonist has been developed and could prove useful in testing whether it can reverse chronic mechanical allodynia [3].

## Central mechanisms implicated in the development of mechanical allodynia

A variety of central mechanisms have been implicated in the establishment of allodynia, including phenotypic changes in peripheral and central neurons as well as an important role for cells of the immune system. Perhaps, the most surprising aspect of nerve damage-related allodynia is the critical role of activated microglia within the central nervous system in this phenomenon, at least in rodent models of these conditions [4]. Sensory neurons are known to play an important role in wound healing through the release of immunoregulatory molecules and mitogens that catalyse the response to injury associated with damaged nerve. One way of thinking about the role of microglia in neuropathic pain is to imagine that the same signals are aberrantly released from seriously damaged nerves both centrally and peripherally, and the recruitment and activation of microglia centrally may be an unfortunate spin-off from the normal neuro-immune interplay involved in wound healing in the periphery. Activation of the ionotropic ATP receptor P2X4 expressed by microglia leads to the release of mediators such as brain-derived neurotrophic factor (BDNF) that can alter chloride transport in the terminals of sensory neurons and dorsal horn neurons, leading to altered patterns of excitability that have been linked to allodynia [4, 38]. Recent evidence suggests that diabetic neuropathy leading to allodynia also involves microglia in animal models [41].

A surprisingly large number of insults seems to be able to induce mechanical allodynia, including peripheral activation of channels that are known to be mechanically insensitive, such as the heat-sensor TRPV1 [31]. Intriguingly, many peptide mediators associated with small-diameter sensory neurons [calcitonin gene-related peptide (CGRP), substance P, BDNF], as well as chemokines [25] have been shown to evoke allodynia when applied intrathecally. Thus, a major afferent barrage from peptidergic nociceptive neurons may sensitise dorsal horn neurons that are normally wired for mechanosensitive input through the possible extra-synaptic actions of these mediators. Specific antagonists of TRPV1 can block mechanical allodynia, suggesting that presynaptic TRPV1 activation is an important element in the release of pro-allodynic mediators. Thus, afferent barrage leading to second-order neural depolarization may cause the release of arachidonic acid metabolites such as 9-HODE that amplify the release of neuromodulators in the dorsal horn [31]. In this way, despite the known mechano-insensitivity of peripheral TRPV1, presynaptic amplification of nociceptive inputs through this receptor contributes to mechanical allodynia.

In the dorsal horn of the spinal cord, the increased peripheral input from sensitised primary afferent fibres also results in TRPV1 activation in GABAergic interneurons. TRPV1 opening increases the intracellular calcium concentration which, if prolonged, will result in long-term depression of these inhibitory interneurons. The GABAergic inhibition exerted on the spinothalamic tract will be released, resulting in enhanced nociceptive inputs to the thalamus [19]. Long-term potentiation of spinal nociceptive neurons and long term depression of inhibitory interneurons together may contribute to setting a greater ongoing activity of dorsal horn neurons and a to the increase of the response intensity to noxious mechanical stimuli. Another consequence of glycinergic inhibition removal is the development of a crosstalk between separate spinal laminae that may be responsible for the rerouting of sensory inputs within the spinal cord. It has been shown, for example, that in response to the pharmacological removal of gycinergic inhibition in the trigeminal ganglion, a local dormant circuit involving PKCγ interneurons is activated, resulting in the gating of tactile inputs to superficial nociceptive specific neurons, turning touch into pain [28]. Nociceptive specific neurons of the spinal cord can furthermore be sensitised through the phenomenon of wind-up (a type of LTP) through the release of substance P by myelinated primary afferent fibres [33]. There is also evidence that myelinated afferent fibres also undergo phenotypic changes that may contribute to the appearance and maintenance of mechanical allodynia in, for example, the expression of potassium channels [37].

## Conclusions and future prospects

Strategies for dealing with allodynia fall into four broad categories. First, identifying and blocking the mediators that sensitise mechanosensory neurons within the peripheral and central nervous system has had some success. In particular, a number of mediators released from activated microglia such as BDNF, as well as various cytokines and NGF all play a role in regulating mechanosensitivity and are potential targets. Secondly, blocking the mechanotransducing channels themselves may be attractive when we finally have a complete list of the molecules involved. As yet, only Piezo2 has been shown to have a potential role in allodynia. Thirdly, attacking electrical excitability in the peripheral neurons that are implicated in allodynia may be useful. A number of pharmacological treatments have been described that diminish allodynia in animal models through sodium channel block, including conotoxin sodium channel-selective blockers that target Nav1.7 and Nav1.8 and limits mechanical hyperalgesia and allodynia [11]. Finally, dismantling the aberrant circuitry that has been linked to allodynia is a potential, if exceptionally complex approach to the problem. It is clear that allodynia results from peripheral drive involving subsets of neurons that are not classical nociceptors, and the sympathetic nervous system plays an important role in some allodynic syndromes. However, there is much basic information that is still lacking, for example, the mechanistic relationship between cold and mechanical allodynia. In particular, a genetic analysis of the role of subsets of A-fibre associated sensory neurons in neuropathic allodynia would be a useful first step in unravelling the circuitry that is involved in the establishment of this unpleasant condition.
